# Effectiveness of DNA-recombinant anti-hepatitis B vaccines in blood donors: a cohort study

**DOI:** 10.1186/1471-2334-7-124

**Published:** 2007-11-06

**Authors:** Emil Kupek, Denise ER de Souza, Andrea Petry

**Affiliations:** 1 National Perinatal Epidemiology Unit, University of Oxford, Old Road Campus, Oxford OX3 7LF, UK; 2 Department of Public Health – Postgraduate Studies Programme, Centre of Health Sciences, Universidade Federal de Santa Catarina, Campus Universitário Trindade, Florianópolis, Brazil; 3 Centre for Hematology and Hemotherapy of the federal state of Santa Catarina, Av. Othon Gama d'Eça 756, Florianópolis, Brazil

## Abstract

**Background:**

Although various studies have demonstrated efficacy of DNA-recombinant anti-hepatitis B vaccines, their effectiveness in health care settings has not been researched adequately. This gap is particularly visible for blood donors, a group of significant importance in the reduction of transfusion-transmitted hepatitis B.

**Methods:**

This is a double cohort study of 1411 repeat blood donors during the period 1998–2002, involving a vaccinated and an unvaccinated cohort, with matching of the two in terms of sex, age and residence. Average follow-up was 3.17 person-years. The outcome measure was infection with hepatitis B virus (HBV), defined by testing positive on serologic markers HBsAg or anti-HBC. All blood donors were from the blood bank in Joaçaba, federal state of Santa Catarina, Brazil.

**Results:**

The cohorts did not differ significantly regarding sex, age and marital status but the vaccinated cohort had higher mean number of blood donations and higher proportion of those residing in the county capital Joaçaba. Hepatitis B incidences per 1000 person-years were zero among vaccinated and 2,33 among non-vaccinated, resulting in 100% vaccine effectiveness with 95% confidence interval from 30,1% to 100%. The number of vaccinated persons necessary to avoid one HBV infection in blood donors was estimated at 429 with 95% confidence interval from 217 to 21422.

**Conclusion:**

The results showed very high effectiveness of DNA-recombinant anti-HBV vaccines in blood donors. Its considerable variation in this study is likely due to the limited follow-up and the influence of confounding factors normally balanced out in efficacy clinical trials.

## Background

There is a wide consensus that vaccination against hepatitis B virus (HBV) is the best cost-effective measure to combat the disease [1]. In Brazil, its burden is grossly underestimated due to the epidemiologic surveillance which captures primarily severe cases in need of hospital treatment while missing the long term consequences of HBV infection such as cirrhoses and hepatocelular carcinoma. It has been estimated that vaccinating children against HBV in developing countries would prevent loss of three million lives each year, as well as 60–80% of cases of hepatocelular carcinoma [1-3]. More than 150 countries have already given high priority to the vaccination programs against HBV [3], but some of them, including Brazil, have not fully implemented such programs.

Brazil has recently started producing a DNA-recombinant anti-HBV vaccine whose immunogenicity has been confirmed in several studies [4-7]. However, its effectiveness in reducing HBV incidence in health care settings has not been adequately researched. This gap is particularly visible regarding blood donors – a group of special importance in preventing HBV transmission and therefore explicitly targeted for immunization at any age according to the guidelines of the Brazilian Ministry of Health [8].

Although the residual risk of not detecting HBV by routine serologic screening in the largest blood bank in the state capital Florianopolis has been reduced considerably in the decade of 1990, it remains at a level approximately hundred times higher than in the developed countries [9,10], thus seriously undermining transfusion recipient safety. Another reason to strongly encourage vaccination among blood donors and verify its effectiveness is recent evidence of a high risk group using blood bank serologic screening to check their HIV status due to the guarantee of obtaining an anonymous, free of charge and rapidly delivered test result in this health setting [10,11].

The aim of this study is to evaluate the effectiveness of DNA-recombinant anti-HBV vaccines among blood donors in an endemic area in Brazil. Although there are no reasons to believe that the vaccine efficacy in blood donors should be much different from that of other healthy adults, its effectiveness may not necessarily follow this logic because it is highly susceptible to selection bias within a particular setting. In the blood donor context, this bias may arise because the main protection factor (getting vaccinated) is likely to be influenced by a more general attitude towards health which also reduces risk behavior for HBV infection. Differently from a randomized controlled trial of vaccine efficacy, it would be ethically unacceptable to randomly allocate anti-HBV vaccine to blood donors because its protective effect has already been proven beyond any doubt. It is therefore necessary to make post hoc adjustment for risk factor imbalances between vaccinated and non-vaccinated blood donors in order to evaluate the benefit of the vaccine achieved in a particular health care setting, i.e. the vaccine effectiveness. The results can then be compared to those achieved in randomized controlled trials of vaccine efficacy in healthy adults which can be considered an upper bound for vaccine effectiveness.

## Methods

A retrospective double-cohort study [12] was used to estimate the protective effect of anti-HBV vaccination by comparing the incidence of HBV infection in the vaccinated and the non-vaccinated cohort of repeat blood donors. For the purpose of this study, case definition of HBV infection was the presence of either of the two serologic markers used for blood donor screening, namely HBsAg (produced by "Hepanostika-Biomerieux") or anti-HBc IgM+IgG (produced by "Ortho Diagnostics"), confirmed by at least one positive test result on subsequent serologic testing with the same markers. The cohorts were matched by sex, age and municipality of residence.

The vaccinated cohort was recruited from an earlier study on seroconversion [7]. The inclusion criteria for both cohorts were having done at least two blood donations during the period of the study (1998–2002) and being between 18 and 65 years of age. For the vaccinated cohort, an additional requirement was that the blood donors seroconverted after three doses of anti-HBV DNA-recombinant vaccine ("Engerix-B^®^" – SmithKline Beecham Biologicals; "Euvax-B^®^" – LG Chemical, Korea; "ButaNG^®^" – Instituto Butantã, São Paulo, Brazil), with the second and third dose following one and six months after the first dose, respectively. The seroconversion criterion was obtaining the titre of at least 10 UI/l of antibody to HBsAg (test anti-Hbs produced by "Biomerieux") three months after receiving the last dose of the vaccine.

For each eligible vaccinated donor, non-vaccinated donors were looked after within the same age band (18–29, 30–44, 45–65 years), of the same sex, and in the same municipality. If no sex by age pair could be found in the same municipality, an adjacent municipality of residence was searched to obtain this matching. All blood donations were realized in the blood bank in Joaçaba, a county capital situated in an area endemic with hepatitis B.

For the vaccinated donors, the individual contribution to person-time incidence denominator was calculated as the difference between the date of last blood donation and the date last dose of anti-HBV vaccine was applied. For the non-vaccinated donors, the individual person-time contribution was the difference between the dates of the last and the first donation during the study period. If HBV infection occurred, person-time denominators were reduced by half-time between the positive serologic test result and the last seronegative donation [13].

The sample size calculation was based on the incidence ratio of 19:1 for the non-vaccinated against the vaccinated cohort, derived from the reported anti-HBV vaccine efficacy of approximately 95% (against remaining 5% in the same group), the HBV incidence in the non-vaccinated blood donors of 0.67% in the Joaçaba blood bank [14], and errors type I and II of 5% and 20%, respectively. Assuming these parameters and equal person-time denominator for both cohorts, Breslow-Day method resulted in 2148 person-years and at least 4 HBV cases between the cohorts [15].

Statistical analysis used Stata [16] and WINPEPI [15], with mid-P method to calculate the 95% confidence intervals (CI) and Pearson's chi-square test for significance of the differences between the blood donors' baseline characteristics.

## Results

Total follow-up time of 1411 study participants was 4472 person-years, with average follow-up of 3.17 person-years for all repeat donors and 2.42 and 3.94 person-years for non-vaccinated and vaccinated donors, respectively.

### Baseline differences between cohorts

The distribution of risk factors for HBV was similar among vaccinated donors included and excluded from the analysis of vaccine effectiveness, except for larger percentages of donors with frequent donations prior to the study (chi-square of 535 with 3 degrees of freedom and p < 0.01) and those residing in the county capital (chi-square of 91 with 2 degrees of freedom and p < 0.01) (Table 1).

**Table 1:** Blood donor characteristics regarding main confounding factors, vaccination and inclusion criteria

Risk factors		Included in the analysis				Excluded from the analysis			
Variables	Categories	Non-vaccinated repeat donors		Vaccinated repeat donors		Vaccinated non-repeat donors		Vaccinated donors lost to follow-up	
		
		n	%	n	%	n	%	n	%

Age bands (years)	18–29	112	15.7	105	15.0	26	12.7	18	16.4
	30–44	351	49.2	323	46.3	106	52.0	56	50.9
	45+	250	35.1	270	38.7	72	35.3	36	32.7
Sex	Masculine	376	52.7	377	54.0	113	55.4	51	46.4
	Feminine	337	47.3	321	46.0	91	44.6	59	53.6
Marrital status	Single	169	23.7	179	25.6	5	20.8	40	36.4
	Married	484	67.9	470	67.3	16	66.7	67	60.9
	Separated	12	1.7	13	1.9	1	4.2	2	1.8
	Widowed	24	3.4	19	2.7	2	8.33	1	0.9
	Other	24	3.6	17	2.4	0	0.0	0	0.0
Municipality of residence	County capital	269	37.7	392	56.2	78	38.2	40	36.4
	Other cities *	175	24.5	51	7.3	31	15.2	20	18.2
	Mainly rural **	269	37.7	255	36.5	95	46.6	50	45.4
Number of donations prior to study	1–2	120	16.8	40	5.7	8	33.3	59	54.6
	3–5	403	56.5	89	12.7	13	54.2	34	31.5
	6–10	169	23.7	224	32.1	2	8.33	9	8.33
	>10	21	2.9	345	49.4	1	4.2	6	5.6

* with at least 20,000 inhabitants

** less than 20,000 inhabitants

Among repeat donors included in the analysis of vaccine effectiveness, it is worth noticing a higher percentage of vaccinated (56.2%) than non-vaccinated (37.7%) residing in the county capital, as well as a higher number of frequent donors among the former compared to the latter (Table 1). Consequently, average interval between donations was significantly longer among the non-vaccinated (0.62 years, 95% CI from 0.58 to 0.65 years) compared to the vaccinated donors (0.46 years, 95% CI from 0.43 to 0.50 years).

### Anti-HBV vaccine effectiveness

Anti-HBV vaccine effectiveness was calculated as one minus the incidence ratio of vaccinated to non-vaccinated donors. The vaccine was 100% effective, with 95% CI from 30.1% to 100% (Table 2).

**Table 2:** Effectiveness of anti-HBV vaccine, incidence ratio (IR) and 95% confidence interval (CI)

Cohort	HBV cases	Total follow-up (person-years)	Person-years at risk*	Incidence per thousand person-years	IR (95% CI)
Vaccinated	0	2747	2715	0.00	0 (0.000, 0.696)
Non-vaccinated	4	1725	1717	2.33	1.000 **

* see methods section for calculation details

** reference category

The difference in HBV incidence between the cohorts compared was 2.33 (95% CI from 0.05 to 4.62) per thousand person-years. From the perspective of the number necessary to treat (NNT), it would be necessary to vaccinate 429 (95% CI from 217 to 21422) blood donors to avoid one HBV infection in this population.

In order to verify possible confounding due to the two HBV risk factors found to be significantly different between the cohorts compared, a stratified analysis crossing all levels of these two factors was performed. This resulted in 12 strata (4 levels of the number of prior blood donations crossed with 3 levels of the residence area). No statistically significant difference (p < 0.05) of the difference in HBV incidence between the vaccinated and the non-vaccinated cohort was found either within any of the 12 strata analysed separately or for the Maentel-Hanzel pooled estimate (mean difference -9.82 with 95% CI from -21.19 to 1.54).

## Discussion

The results of this study showed excellent effectiveness of DNA-recombinant anti-HBV vaccines in blood donors, similarly to the results in the general population [17-20]. Wide confidence intervals for the vaccine effectiveness are probably due to the very small numbers in the incidence numerators natural for rare events and presence of other factors influencing the vaccine effectiveness in a health care setting which are better controlled for or balanced out in a clinical trial setting.

The countries that implemented a large scale vaccination against HBV and maintained it for at least a decade managed to reduce the HBV incidence more than ten times during this period [17-20]. Brazilian Ministry of Health has recently introduced a similar anti-HBV vaccination program with mandatory vaccination for children and adolescents up to 15 years of age [8]. However, only children in the first year of life have had reasonable vaccine coverage, thus leaving a vast majority of the population, including blood donors, unprotected against HBV. A catch-up vaccination campaign would be appropriate to accelerate the reduction of HBV in the general population. In addition, Brazilian blood donors should be systematically encouraged to get vaccinated with DNA-recombinant anti-hepatitis B vaccine. Although this group has been clearly emphasised in the recent vaccination guidelines [8], the logistics necessary to meet this target have not been reinforced nationwide, so it is basically up to a blood donor to seek a primary care unit and solicitate anti-HBV vaccination on individual basis.

Several limitations of this study should be born in mind. First, although matching by sex and age has likely removed major confounding factors and the stratified analysis confirmed the main results of the study, it is still possible that residual confounding factors such as motivation to donate blood have not been fully accounted for in the analysis. For example, the motivation to donate blood repeatedly is known to be associated with lower chances of being infected with HBV compared to first-time blood donors. A prospective cohort study would have better means of controlling for this factor. Second, the absence of HBsAg positive result in the early phase of HBV infection may be as high as 25% [13], thus leaving a period of several months before anti-HBC becomes detectable by routine serologic testing in blood banks for a considerable proportion of donors. During this period, neither of these two HBV markers can detect the virus, leading to underestimation of the true HBV incidence. Third, very few cases of HBV seroconversion, as well as the fact that all of them occurred in the non-vaccinated cohort, reduced the statistical power to estimate vaccine effectiveness with precision, thus leading to wide confidence intervals. Small number of the events of interest was also prohibitive for the applicability of time-to-event multivariate regression methods, capable of a more precise statistical adjustment for relevant covariates than the univariate matched group analysis used in this study. Fourth, the blood donors resided in an endemic area for HBV where the infection rate in adult population is likely to be high, as indicated by the HBV incidence of 2.33 per thousand person-years observed in the non-vaccinated cohort. An incidence of this order could be significantly reduced by vaccination compared to the impact of vaccination in low endemic areas, therefore limiting the generalisability of anti-HBV vaccine effectiveness from this study to such areas.

Despite the limitations, this is the first study of anti-HBV vaccine effectiveness in blood donors using a cohort study with matched control group to minimize the impact of confounding factors. The same methodology can be applied to other blood banks without interfering with their daily routine. A multi-centre evaluation of anti-HBV vaccine effectiveness can be set up on a regular basis with this study design, providing important information about the reduction of HBV infection in the adult population.

In a wider perspective, it should be borne in mind that the results of this study have demonstrated medium term protective effects of DNA-recombinant anti-HBV vaccines in blood donors as means of reducing the transfusion-transmitted HBV in Brazil. At present, long term benefits of a widespread anti-HBV vaccination in Brazil cannot be evaluated because vast majority of the vaccinees have not yet reached the minimum age for blood donation (18 years). As government-sponsored mass vaccination against HBV has started approximately a decade ago and has so far achieved reasonable vaccine coverage only for the children in the first year of life, blood donor population in Brazil continues exposed to HBV. This underlies the need for targeted vaccination of blood donors in addition to the children and adolescent vaccination.

## Conclusion

The results showed a very high effectiveness of DNA-recombinant anti-hepatitis B vaccination among blood donors resulting in the enhancement of blood recipient safety in the State of Santa Catarina. Considerable variation of this estimate is likely due to the limited follow-up and the influence of confounding factors normally balanced out in efficacy clinical trials.

## Competing interests

The author(s) declare that they have no competing interests.

## Authors' contributions

Emil Kupek and Denise E. R. de Souza designed the study. Denise E. R. de Souza and Andrea Petry collected the data. Emil Kupek did the statistical analysis and wrote the draft of the paper. All authors contributed to the writing of the final version of the paper.

## Pre-publication history

The pre-publication history for this paper can be accessed here:


